# Antagonizing IL-6 receptor restores pancreatic tissue resident NK cells activation and ameliorates pancreatic injury in the mouse model of MASH

**DOI:** 10.3389/fphar.2025.1611637

**Published:** 2025-07-07

**Authors:** Johnny Amer, Diana Abu Arra, Ahmad Salhab, Faris Kayed, Muneer Maali, Raghad Shweiki, Mustafa Ghanim

**Affiliations:** ^1^ Department of Allied and Applied Medical Sciences, Faculty of Medicine and Allied Medical Sciences, An-Najah National University, Nablus, Palestine; ^2^ Department of Biomedical Sciences and Basic Clinical Skills, Faculty of Medicine and Allied Medical Sciences, An-Najah National University, Nablus, Palestine

**Keywords:** pancreatic injury, pancreatic fibrosis, trNK cells, IL-6, IL-6R, MASH

## Abstract

**Background and aim:**

Metabolic-associated steatohepatitis (MASH) and pancreatic inflammation are key complications of obesity-related metabolic syndrome. Elevated IL-6; a proinflammatory cytokine, contributes to liver steatosis and pancreatic β-islet cells dysfunction. This study explores pancreatic tissue-resident (tr)NK cells IL-6 receptor (IL-6R) in pancreatic injury in a murine MASH model.

**Methodology:**

MASH models were established using male *Ob/Ob* mice fed a high-fat diet (*Ob/Ob* ^HFD^; 60.3% kcal from fat) for 4 weeks and using immunocompromised NOD-SCID IL2rγnull (NSG) mice fed with HFD for 16 weeks and *i.v.* injected with 10 × 10^6^ pancreatic trNK and treated with IL-6R antagonizing antibody on week 12. Biochemical assays assessed serum ALT, AST, lipids, glucose, and insulin levels. Pancreatic injury was analyzed through mRNA expression of Reg1, Reg3, oxidative stress marker of tissue malondialdehyde (MDA) and β−islet cells’ proliferation and apoptosis. Fibrotic markers of α-SMA, Collagen-I, and Fibronectin were assessed via RT-PCR and trNK cell activation (CD107a, NKp46, IFN-γ) were assessed by flow cytometry.

**Results:**

*Ob/Ob* ^HFD^ mice exhibited increased serum cholesterol, triglycerides, fasting blood glucose, and liver injury enzymes. Markers of pancreatic injury of Reg1/Reg3 and pancreatic MDA with β−islet cells apoptosis were significantly elevated compared to littermates’ control. These results were accompanied by a decline in trNK counts and activations (P < 0.05). In an adoptive transfer model, NSG mice fed with HFD and transplanted with trNK cells from *Ob/Ob* ^HFD^ donors (expressing high IL-6) exhibited similar pancreatic injury markers, whereas those receiving trNK cells from *Ob/Ob* ^HFD^ mice pre-treated with an IL-6R antagonist showed marked reductions in Reg1/Reg3 (∼2-fold), MDA (∼1.77-fold), and β-islet cells apoptosis (∼2.2-fold). Moreover, phenotypic characterization of the NSG mice fed an HFD transplanted with IL-6R antagonizing antibody showed an increase in the NK cell activation marker CD107a (∼2.3-fold) and amelioration in pancreatic fibrotic profile of α-SMA mRNA expressions of 1.6 -fold when compared to its counterparts.

**Conclusion:**

Our data highlights the importance of IL-6R modulation on trNK cells in remodeling pancreatic tissue after liver injury, emphasizing the liver-pancreas axis as a therapeutic target to prevent pancreatic damage, β-islet cells dysfunction and fibrosis and reduce the risk of diabetes and metabolic syndrome.

## Introduction

Obesity is a complex, multifactorial disease characterized by excessive adipose tissue accumulation and chronic low-grade inflammation, which together contribute to widespread metabolic, hormonal, and immunological disturbances across multiple organ systems (Bluher, 2019). While liver complications, such as metabolic-associated fatty liver disease (MAFLD), are well-established, recent evidence highlights the pancreas as a secondary but highly susceptible organ affected by obesity-induced inflammation (Leslie et al., 2020). Chronic inflammatory signaling, driven by adipose tissue-derived cytokines and dysregulated lipid metabolism, creates a systemic environment conducive to pancreatic dysfunction (Wang et al., 2021). Notably, the accumulation of ectopic fat within and surrounding the pancreas, referred to as pancreatic steatosis, has been implicated in impaired insulin secretion, increased risk of type 2 diabetes, and heightened vulnerability to pancreatitis (Petrov and Yadav, 2019). Moreover, Natural killer (NK) cells, an important player in immune surveillance and tissue homeostasis, may become functionally impaired under obesity-induced systemic inflammation or acute pancreatic stress conditions (Paul and Lal, 2017).

Among the major key inflammatory mediators, interleukin-6 (IL-6) has gained attention for its dual role in propagating inflammation and serving as a clinical biomarker (Tanaka et al., 2014). Elevated serum IL-6 levels are commonly observed in individuals with acute pancreatitis and correlate with disease severity and adverse outcomes (Rao and Kunte, 2017). In addition to its diagnostic relevance, IL-6 has emerged as a potential therapeutic target; preclinical studies demonstrate that pharmacologic reduction of IL-6 levels using agents such as carnosine or artemether can significantly attenuate pancreatic inflammation and injury (Benchikh et al., 2024). However, IL-6 also performs essential physiological functions, including regulating immune responses, acute-phase protein production, and tissue regeneration (Hirano, 2021). As a result, systemic inhibition of IL-6, such as through monoclonal antibodies like tocilizumab, has been associated with increased susceptibility to infection, impaired liver function, and delayed wound healing, particularly in chronic treatment settings (LiverTox: Clinical and Research Information on Drug-Induced Liver Injury, 2012). These findings underscore the need for selective, tissue-specific approaches to modulating IL-6R on pancreatic trNK cells in order to mitigate inflammation without compromising host immunity or homeostatic repair mechanisms, which was our primary aim in the current study. Additionally, we sought to elucidate the immune-metabolic axis between the liver and pancreas, providing insights into inter-organ inflammatory signaling and its contribution to modulating obesity associated with metabolic diseases.

## Methods

### Animal model

Twelve-week-old male leptin-deficient mice (*Ob/Ob*) and their littermates (n = 10 in each group) were obtained from Envigo (Harlan). To model MASH, two experimental mouse models were utilized. In the first model, *Ob/Ob* mice were fed a high-fat diet (HFD) for 4 weeks to induce hepatic and metabolic injury (*Ob/Ob* ^HFD^). The diet used (Cat. # TD.06414) consisted of 60.3% kcal from fat, 21.4% from carbohydrates, and 18.3% from protein, replicating a lipotoxic nutritional environment. In the second model, immunodeficient NOD-SCID IL2rγ^null^ (NSG) mice, which lack functional T, B, and NK cells, were fed the same HFD for 16 weeks starting at 10 weeks of age. In week 12, these recipient NSG mice were *i.v*. injected with 10 × 10^6^ pancreatic tissue-resident (tr)NK isolated from *Ob/Ob* ^*HFD,*^ which were pre-treated with IL-6R antagonizing antibody (10 μg/10^6^ cells) or IgG-isotype control. The cell injection was administered twice a week for the remaining 4 weeks (n = 6 in each group). *Ob/Ob* ^*HFD*^ without adoptive transfer (untreated with trNK cells were used as a control. Mice received care in accordance with the ethical regulations of An-Najah National University (ANNU) and the NIH guidelines (Ref: Mas. May 2022/15). To minimize animal suffering, mice were monitored daily for signs of distress, weight loss, reduced activity, or abnormal grooming. Humane endpoint criteria were pre-defined, including a weight loss of more than 20%, significant behavioral changes, or signs of pain unresponsive to supportive care. All surgical procedures and sample collections were performed under anesthesia using 5% isoflurane inhalation followed by cervical dislocation to ensure rapid and humane euthanasia. Efforts were made to reduce the number of animals used by applying the principles of the 3Rs (Replacement, Reduction, and Refinement). Group sizes were based on minimal requirements to obtain statistically meaningful results while minimizing unnecessary animal use.

### Serum metabolic profile assessments

On the sacrifice day, whole blood was withdrawn through cardiac puncture and collected in heparinized tubes for isolating serum (blood was centrifuged at 3,500 rpm for 30 min at 4°C) and assessed by ELISA for liver injury enzymes and biochemical assessment as described below. The following laboratory measurements were obtained: serum ALT (Abcam; ab285263), AST (Abcam; ab263882), fasting blood sugar (Biocompare; MBS7200879), insulin (Abcam; ab277390), cholesterol (Abcam; ab285242), and triglycerides (Biocompare; MBS726589). In accordance with standard laboratory protocol, it was ensured that all reagents and samples were equilibrated to room temperature (18°C–25°C) before their use. A 100 µL volume of each standard and sample was added to the respective wells and incubated for 2.5 h at room temperature with moderate shaking. After incubation, the solution was discarded, and wells were washed four times with 1X Wash Solution. It is worth noting that the washing process involved filling each well with Wash Buffer (300 µL), using a multi-channel pipette or auto washer. Ensuring the complete liquid removal at each step is crucial for optimal performance. A 100 µL of 1x prepared detection antibody was added to each well for 1 h at room temperature with gentle shaking. Following this, 100 µL of the prepared streptavidin solution was added to each well and incubated for 45 min at room temperature with gentle shaking. A volume of 100 µL of TMB One-Step Substrate Reagent (Item H) was added to each well for 30 min at room temperature in the dark with gentle shaking. Finally, 50 µL of Stop Solution (Item I) was added to each well. Absorbance was immediately read at 450 nm using an ELISA reader (Tecan M100 Plate Reader).

### Luminex MAGPIX tests

Multiplexed sandwich enzyme-linked immunosorbent assay-based technology (Cat# MHSTCMAG-70K; R&D Systems) was used to simultaneously determine the concentrations of multiple cytokines (IL-6, IL-1β, and IL-8). Samples from each mouse were analyzed as described in the manufacturer’s instructions (Mateu-Borrás et al., 2025). Briefly, samples and standards were diluted and incubated overnight at 4°C with a premixed cocktail of fluorescent-coded magnetic beads, each conjugated to a specific capture antibody. The following day, plates were washed three times using a handheld magnetic separator and incubated sequentially with a biotinylated detection antibody cocktail for 1 h at room temperature, followed by streptavidin–phycoerythrin (PE) for 30 min. After a final series of washes, the beads were resuspended in assay buffer and analyzed using a Luminex MAGPIX system. Cytokine concentrations were calculated based on standard curves generated from known concentrations using xPONENT^®^ software. All samples were run in duplicate to ensure reproducibility, and intra-assay variation was monitored using quality control beads provided with the kit.

### RNA isolation, cDNA preparation, and real-time PCR

The liver and pancreas tissues were subjected to RNA isolation using 2 mL of TRI Reagent (Bio-Lab; Cat# 90102331) per cm^3^ of tissue. The samples were homogenized for 5 min at room temperature before adding 0.2 mL of chloroform (Bio-Lab; Cat# 03080521). Following a 15-min incubation period at an ambient temperature, the samples were centrifuged at 1,400 revolutions per minute for 15 min at 4°C. The supernatant from each sample was carefully transferred to a fresh micro-centrifuge tube during RNA precipitation. After adding 0.5 mL of isopropanol (Bio-Lab; Cat# 16260521), the mixture was incubated at 25°C for 10 min. The tubes were then centrifuged (12,000 rpm) for 10 min at 4°C, the supernatants were removed, and 1 mL of 75% ethanol was added to the pellet. Following centrifugation at 7,500 revolutions per minute (rpm) for 5 min, the resulting pellets were subjected to air drying at ambient temperature for 15 min. After adding 50 μL of DEPC, the samples were thermally treated at 55°C for 10 min. The R&D High-Capacity cDNA Isolation Kit (1406197) was used to produce cDNA. The αSMA and collagen I, Fibronectin, Reg1, and Reg3 gene expressions were quantified using real-time PCR with the TaqMan Fast Advanced Master Mix (Applied Biosystems; Cat# 4371130). The primers and probes used for RT-PCR targeting α-SMA, Collagen I, Fibronectin, Reg1, and Reg3 were commercially purchased as pre-validated TaqMan^®^ Gene Expression Assays from Rhenium (Israel), an authorized distributor of Thermo Fisher Scientific. These assays are proprietary and patented; therefore, the exact primer and probe sequences are not disclosed by the manufacturer. However, the use of TaqMan^®^ chemistry ensures high specificity and efficiency, as the assays are designed and optimized based on exon-exon junctions and genomic context using Thermo Fisher’s validated bioinformatics pipeline. Gene expression was normalized to that of the housekeeping gene GAPDH. In the first experimental model involving Ob/ObHFD mice, gene expression data were normalized and expressed as fold changes relative to the littermate control group, which was assigned a baseline value of 1. However, in the second adoptive transfer model, NSG mice fed an HFD without pancreatic trNK cell administration were used as the control group. The cycling conditions for the one-step RT-PCR involved 40 cycles of 94°C for 30 s, 60°C for 30 s, and 72°C for 1 min, followed by a final extension at 72°C for 10 min. The data were analyzed using a QuantStudio™ 5 Real-Time PCR System (Cat# A34322, Applied Biosystems).

### β-Islet cells isolation

Pancreatic β−islet cells isolation was performed using a standard collagenase digestion method followed by density gradient purification. Briefly, the pancreas was perfused via the common bile duct with a collagenase solution (typically Collagenase P or Liberase TL), excised, and incubated at 37°C for 15–20 min to enzymatically dissociate the tissue. The digested tissue was then washed and subjected to a Ficoll-based or Histopaque density gradient to separate intact β-islet cells from exocrine debris. Purified islets were hand-picked under a stereomicroscope and subsequently cultured in RPMI 1640 medium supplemented with 10% fetal bovine serum (FBS), 100 U/mL penicillin, and 100 μg/mL streptomycin at 37°C and 5% CO_2_ until further use.

### Pancreatic oxidant activity assay

The homogenized pancreas tissue was centrifuged at 9,000 *g* for 15 min. The separated supernatant was used to assess oxidative stress through the measurement of Malondialdehyde (MDA), which was determined using an ELISA kit according to the manufacturer’s instructions.

### Pancreatic trNK cells isolation

Under deep ether anesthesia, mice were euthanized using isoflurane, USP 100% (INH). The pancreases were then removed, and a portion of them was transferred to a Petri dish containing 5 mL of DMEM medium (Biological Industries; Cat# 01-055-1A). The pancreas tissues were thoroughly dissected using a stainless-steel mesh. The cells were then harvested in the medium and added to 50 mL tubes containing 10 mL DMEM. The cells were subsequently transferred to new tubes containing Ficoll (Abcam; Cat# AB18115269). Tubes were centrifuged for 20 min at 1,600 rpm at 20°C. The supernatant in each tube was transferred to a new tube for a second centrifugation, this time for 10 min at 1,600 rpm and 4°C. After the second centrifugation, the pellet in each tube was resuspended in 1 mL of DMEM for the NK isolation kit (Stem Cells; Cat# 19665). As mentioned below, pancreatic trNK cells were phenotyped as CD45^+^CD3^−^NK1.1^+^CD49a^+^DX5^-^ by flow cytometry. Before flow cytometry analysis, cells (10 (Tanaka et al., 2014)/100 μμΛ) were assessed for their viability, measured by propidium iodide (PI) (A35110, R&D Systems). PI-negative cells were considered viable, with a mean viability rate of 92.7% ± 1.5%.

### Flow cytometry

All antibodies were incubated with the isolated trNK cells suspensions (1:100) at 4°C for 45 min. The cells were washed twice with PBS supplemented with 1% fetal calf serum (FCS), and a secondary antibody (1:100) was added for 45 min at 4°C. The primary mouse antibodies used were anti-CD3 (PE-CY7, BD Bioscience Cat# 560591), anti-NK1.1 (Percpcy-5; Abcam Cat# ab210337), anti-CD49a (PE; BD Bioscience Cat# 559596), anti-DX5 (APC; BD Bioscience Cat# 560591), anti-CD4 (FITC; Cat# ab269349, abcam), anti-CD8 (Percpcy-5; Cat# ab217334, abcam), anti-CD107a (lysosomal-associated membrane protein-1 (LAMP-1) (Pacific blue; BD Bioscience Cat# 564348), anti-NKp46 (FITC; BD Bioscience Cat# 560756), anti-IFN-γ (ab40465, abcam) and IL-6R (ab40465, abcam). The secondary antibodies used were goat anti-mouse IgG (AF488) (ab150113, Abcam) and goat anti-rabbit IgG (APC) (Thermo Fisher, Cat# A10931). Isotype IgG labeled with the relevant fluorochrome was used as a control for each antibody. Apoptosis was evaluated with annexin-V (A35110, R&D Systems) staining. Early apoptotic cells were defined as annexin-V^+^PI^−^ cells, and late apoptotic cells were defined as annexin-V + PI + cells. All stained cells were examined using a flow cytometer (BD LSR Fortessa, Becton Dickinson, Immunofluorometry Systems) and analyzed with FCS Express 7 by *De Novo* Software for flow cytometry.

### Ki-67-FITC staining protocol for β-islet cells

β-Islet cells were harvested and fixed in 70% ethanol at −20°C for at least 2 h to ensure effective permeabilization. Following fixation, cells were washed with PBS and incubated with 0.1% Triton X-100 in PBS for 10 min at room temperature to permeabilize the nuclear membrane. After an additional wash, cells were incubated with an anti-Ki-67 monoclonal antibody (abcam; ab281847) directly conjugated to fluorescein isothiocyanate (FITC) at a 1:100 dilution in PBS with 1% BSA for 30–60 min in the dark at room temperature. Subsequently, samples were washed thoroughly and analyzed using flow cytometry, as mentioned above. A parallel sample stained with isotype-matched FITC-conjugated IgG served as a negative control.

### Western blot analysis

Protein was extracted from pancreatic trNK cells using lysis buffer (50 mmol/L Tris-HCl (pH 7.6), 0.25% Triton X-100, 0.15 M NaCl, and 10 mM CaCl2) and complete Mini EDTA-Free Protease Inhibitor Cocktail (Roche Diagnostics, Mannheim, Germany). Protein concentrations were determined using a BCA protein assay kit (Thermo Fisher Scientific, Waltham, MA, United States). The obtained proteins were then mixed with 5× loading buffer (protein: 5× loading buffer = 4:1) and boiled at 95°C for 10 min. Next, 20 μg of protein was separated per lane on a 10% (w/v) sodium dodecyl sulfate (SDS)-polyacrylamide gel (Bio-Rad) under reducing conditions. Proteins were transferred to a polyvinylidene fluoride (PVDF) membrane for immunoblotting. The membranes were then incubated for 1 h at room temperature in neutralizing buffer containing 5% skim milk (w/v) before being incubated with rabbit anti-human/mouse p-STAT3 (S727) (AF4934, R&D Systems) and anti-human/mouse STAT-3 (MAB1799, R&D Systems). All primary antibodies were diluted 1:1,000 and incubated with the membrane overnight at 4°C. The membranes were incubated with peroxidase-conjugated anti-mouse and anti-rabbit IgG (Abcam, 1:5000 dilution) for 1.5 h at room temperature. Immunoreactivity was detected using an enhanced chemiluminescence kit (Abcam). Imaging and data analysis were performed using a FUSION Solo S system.

### Histological assessment of mouse livers

The posterior third of the mice’s liver was dissected and fixed with 4% formalin for 24 h at room temperature, embedded in paraffin, and sliced (7 μm) in an automated tissue processor (microtome). Sections were deparaffinized by immersion in xylene, as mentioned in Amer et al. (Amer et al., 2022). The sections were then rehydrated using a graded series of ethanol concentrations, starting with absolute ethanol and ending with distilled water. Hematoxylin and eosin (H&E) staining was used to evaluate the level of steatosis, necro-inflammatory regions, and apoptotic bodies. A veterinary pathologist assessed all histopathological findings and reported assessment grades. Stained slides were scanned using a Zeiss microscope.

### Statistical analysis

Statistical differences were analyzed using a two-tailed unpaired Student’s t-test for comparisons between two groups, or two-way analysis of variance (ANOVA) for comparisons among multiple groups, performed with GraphPad Prism 9.0 (GraphPad Software, La Jolla, CA). When ANOVA indicated significant differences, Tukey’s *post hoc* multiple comparisons test was applied to assess pairwise group differences. A p-value ≤0.05 was considered statistically significant. Data are presented as averages ±standard deviation (SD).

## Results

### *Ob/Ob* ^*HFD*^ mice model exhibited a dysfunctional metabolic profile that was associated with liver injury

**FIGURE 1:**
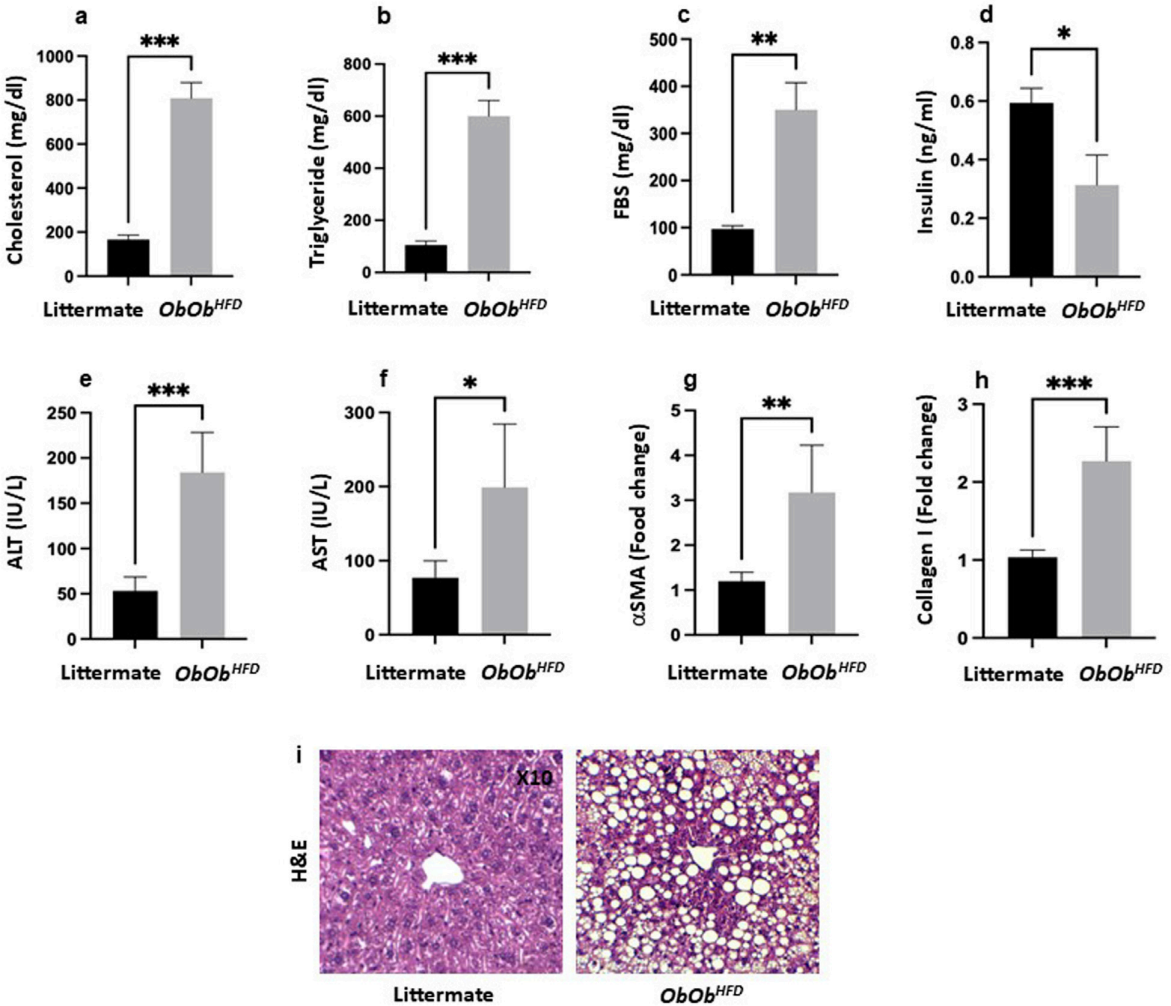
*Ob/Ob* ^*HFD*^ exacerbates increased metabolic and liver injury markers. Serum **(a)** cholesterol (mg/dL), **(b)** triglycerides (mg/dL), **(c)** fasting blood sugar (FBS; mg/dL), **(d)** insulin (ng/mL), **(e)** alanine aminotransferase (ALT; IU/L), **(f)** aspartate aminotransferase (AST; IU/L) were assessed using ELISA. **(g)** α‐smooth muscle actin (αSMA; fold change) and **(h)** collagen I (fold change) were assessed using RT-PCR, and GAPDH was used as a housekeeping gene. **(i)** H&E staining of liver sections (×10 magnification). Black bars represent littermate controls; gray bars represent *Ob/Ob* ^*HFD*^ mice (n = 10) in every group. Data are expressed as averages±SD; *p < 0.05, **p < 0.01, ***p < 0.001 by unpaired two-tailed Student’s t-test.

In the current study, we adapted the *Ob/Ob* mice model to mimic MAFLD. Additionally, we introduced HFD [*Ob/Ob* ^*HFD*^] for 4 weeks to exacerbate systemic fat accumulation in the liver with a possible potential to cause liver fibrosis. To investigate the metabolic involvement in *Ob/Ob* ^*HFD*^ mice, we examined hyperglycemia, hyperlipidemia, and insulin resistance. Serum cholesterol and triglyceride levels were significantly elevated in the *Ob/Ob* ^*HFD*^ mice compared to their naive littermates. Serum cholesterol levels in naive mice were measured at 180 ± 20 mg/dL, while those in the *Ob/Ob* ^*HFD*^ mice were 800 ± 24 mg/dL, representing a four-fold increase (P = 0.001; Figure 1A). In addition, *Ob/Ob* ^*HFD*^ mice exhibited elevated serum triglyceride levels of 600 ± 31 mg/dL, compared to 120 ± 15 mg/dL in their littermate counterparts (P = 0.001), indicating a six-fold increase (Figure 1B). Furthermore, fasting blood sugar (FBS) levels in naive littermates were 100 ± 8 mg/dL, whereas in the *Ob/Ob* ^*HFD*^ mice, they increased to 350 ± 55 mg/dL, reflecting a 3.5-fold increase (Figure 1C). Insulin levels in naive mice were measured at 0.6 ± 0.02 ng/mL, while *Ob/Ob* ^*HFD*^ mice showed a significant 50% reduction, with insulin levels dropping to 0.3 ± 0.1 ng/mL (Figure 1D). This decline in insulin suggests impaired insulin production or function in the *Ob/Ob* ^*HFD*^ mice, further reinforcing their utility as a model for studying diabetes. In summary, this experiment demonstrated that *Ob/Ob* ^*HFD*^ mice developed significant metabolic dysfunction, mirroring the obesity and related metabolic disorders observed in humans. The severe increases in cholesterol, triglycerides, fasting blood glucose, and the reduction in insulin levels support the conclusion that this mouse model effectively represents key aspects of obesity, metabolic syndrome, and Type 2 diabetes.

Lab assessments of liver injury of serum levels of ALT levels were significantly elevated in *Ob/Ob* ^*HFD*^ mice compared to littermate controls (*Ob/Ob* ^*HFD*^: 175 ± 35 IU/L vs. Littermate: 50 ± 12 IU/L, p < 0.001), indicating increased hepatocellular injury (Figure 1E). Similarly, AST levels were markedly increased in *Ob/Ob* ^*HFD*^ mice relative to littermate controls (*Ob/Ob* ^*HFD*^: 200 ± 83 IU/L vs. Littermate: 90 ± 14 IU/L, p < 0.01), further supporting liver damage in the MASH model (Figure 1F). These findings confirm that the *Ob/Ob* ^*HFD*^ mouse model effectively recapitulates key features of MASH, including severe liver steatosis and elevated liver injury enzymes (ALT and AST). Moreover, Quantitative real-time PCR analysis revealed a significant upregulation of key fibrotic genes in the livers of *Ob/Ob* ^*HFD*^ mice compared to their littermate controls. The expression of α-smooth muscle actin (α-SMA) was markedly increased, demonstrating a threefold elevation in *Ob/Ob* ^*HFD*^ (3.0 vs. 1.0; Figure 1G). Similarly, collagen type I alpha 1 (Col1a1) expression was elevated to 2.3-fold relative to control mice (2.3 vs. 1.0, Figure 1H), indicating enhanced extracellular matrix deposition. These transcriptional changes reflect the onset of fibrotic remodeling and support the utility of the *Ob/Ob* ^*HFD*^ model in mimicking liver fibrosis associated with metabolic dysfunction. Histological evaluation using hematoxylin and eosin (H&E) staining revealed notable hepatic architectural disruption in *Ob/Ob* ^*HFD*^ mice compared to their littermate controls. The littermate panel represents a normal liver architecture, characterized by well-organized hepatic cords radiating from the central vein, with polygonal hepatocytes exhibiting round, centrally located nuclei and eosinophilic cytoplasm. There is no evidence of steatosis, inflammation, or fibrosis. In contrast, the *Ob/Ob* ^*HFD*^ panel exhibits marked macrovesicular steatosis, indicating significant lipid accumulation within hepatocytes. The hepatocyte cytoplasm is displaced by large lipid vacuoles, causing the nuclei to be pushed to the periphery. The presence of widespread fat droplets distorts the lobular architecture. No overt inflammatory infiltrate or fibrosis is visible at this magnification, but the steatotic changes are consistent with features of MASH in the appropriate clinical context (Figure 1I). The significant increase in these biomarkers validates the *Ob/Ob* ^*HFD*^ model as a robust tool for studying MASH pathophysiolog.

### *Ob/Ob* ^*HFD*^ mice model showed a pancreatic injury profile via oxidative stress

**FIGURE 2:**
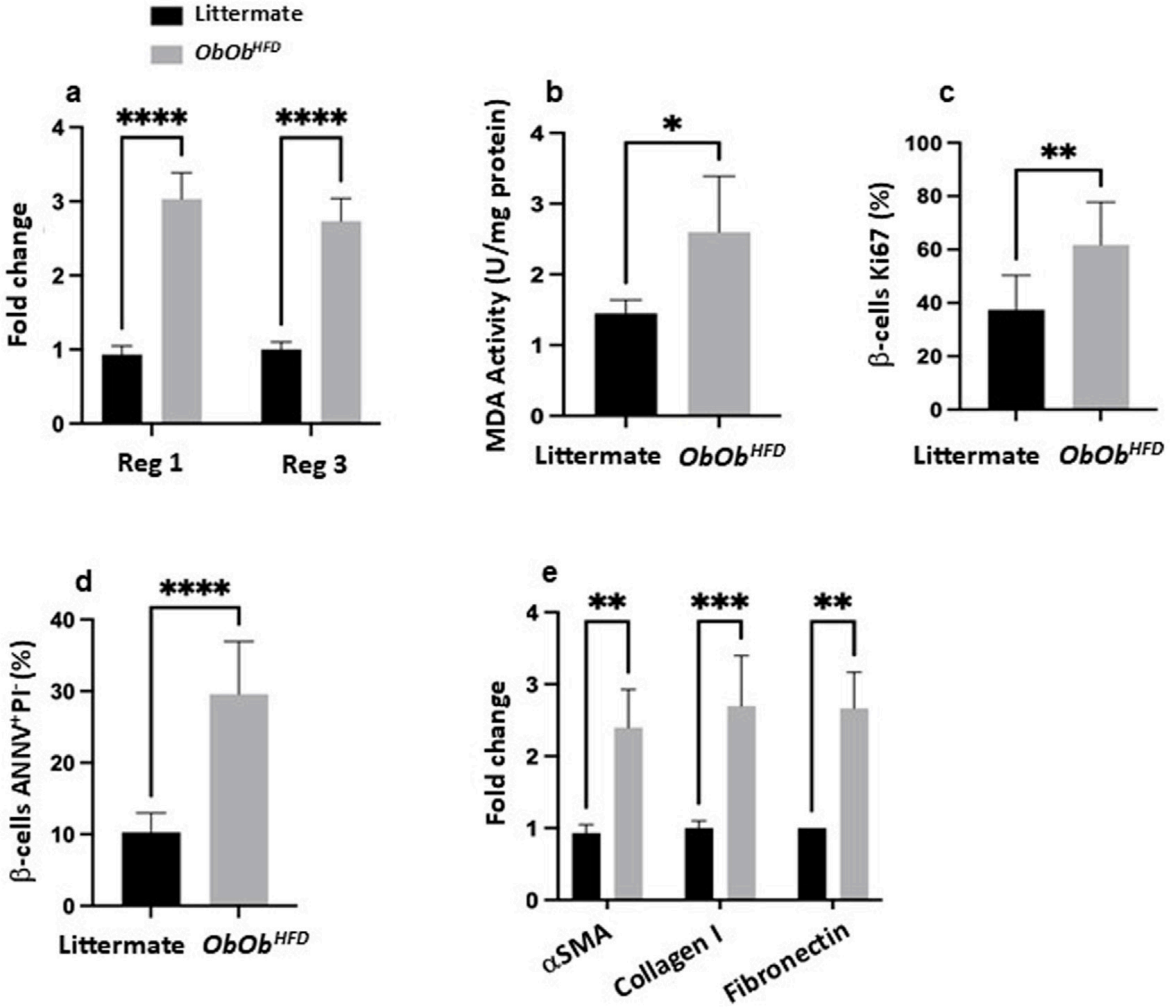
*Ob/Ob* ^*HFD*^ triggers pancreatic stress, β‐cell apoptosis, and fibrotic changes. **(a)** mRNA expression levels of Reg1 and Reg3 (fold change) in pancreatic tissue; **(b)** MDA activity (U/mg), serving as an index of oxidative stress; **(c)** percentage of β‐cells positive for Ki67 (a proliferation marker); **(d)** the percentage of β‐cells undergoing apoptosis, determined by Annexin V and propidium iodide (ANNV^+^/PI^+^) staining; **(e)** mRNA expression levels (fold change) of fibrotic markers, including α‐smooth muscle actin (αSMA), collagen I, and fibronectin. Black bars represent littermate controls; gray bars represent *Ob/Ob* ^*HFD*^ mice (n = 10) in every group. Data are expressed as averages±SD; *p < 0.05, **p < 0.01, ***p < 0.001, ****p < 0.0001 by unpaired two-tailed Student’s t-test.

To assess the impact of liver disease on the extent of pancreatic injury in *Ob/Ob* ^*HFD*^ mice, we first measured the pancreas-to-mouse weight ratio and the expression of the regenerating islet-derived genes Reg1 and Reg3, which serve as sensitive markers of pancreatic injury and attempted repair (Girón-González and Salto, 2021). The pancreas-to-mouse weight ratio demonstrated a notable 1.7-fold increase in the obese mice model compared to the littermate control group (Supplementary Figure S1). This observation suggests a potential regenerative response in the pancreas, possibly due to injuries incurred within the organ. In addition, our experiments showed that *Ob/Ob* ^*HFD*^ mice exhibited a dramatic upregulation of both Reg1 (3.0 ± 0.2 vs. 0.9 ± 0.1 in controls) and Reg3 (2.7 ± 0.3 vs. 1.0 ± 0.1; p < 0.0001 for both; Figure 2A), confirming a heightened regenerative response in the *Ob/Ob* ^*HFD*^ pancreas. Nevertheless, additional tissue injury assessments of oxidative damage were performed via evaluating for MDA activity, a well-established measure of lipid peroxidation. Pancreatic tissue isolated from *Ob/Ob* ^*HFD*^ showed significantly higher MDA levels (2.6 ± 0.4 U/mg) as compared to their littermates’ controls that revealed levels of 1.4 ± 0.2 U/mg; p < 0.05; Figure 2B), emphasizing oxidative stress involvement in the regeneration process. To further investigate the behavioral phenotypic characteristics of β-islet cells, we assessed Ki67 and annexin V to evaluate proliferation and apoptosis, respectively. Figure 2C displayed a marked increase in Ki67 staining of isolated β-islet cells in the *Ob/Ob* ^*HFD*^ (60% ± 18%) as compared to their littermates’ controls that showed staining of 38 % ± 12% (p < 0.01), indicating a highly proliferative cell. In parallel, β-islet cells exhibited a substantial increase in apoptosis rate of 3-fold as compared to littermates’ controls (Figure 2D; p < 0.0001).

We next quantified mRNA levels of α-SMA, Collagen I, and Fibronectin in pancreatic tissue, as a key response to chronic pancreatic damage, which also plays a significant role in the progression of pancreatic diseases, including pancreatitis, pancreatic cancer, and metabolic disorders such as obesity and diabetes (Li X. et al., 2021). Figure 2E illustrates a 2.4 ± 0.6, 2.7 ± 0.6, and 2.6 ± 0.5-fold increase in α-SMA, Collagen I, and Fibronectin expression, respectively, in the *Ob/Ob* ^*HFD*^ mice as compared to littermates’ controls (p < 0.01), reflecting pro-fibrotic activation. Collectively, our results demonstrate pancreatic damage reflected by oxidative injury and compensatory regenerative process with highly proliferative cells, yet β-islet cells showed a low survival rate as the accumulation of ECM and fibrosis in the pancreas is thought to disrupt the microenvironment required for β-cell survival. Studies have shown that fibrotic tissue can limit the nutrient and oxygen supply to the remaining beta cells, leading to additional stress and further increasing the likelihood of apoptosis (Li J. et al., 2021). Furthermore, the altered extracellular environment can impair the regenerative capacity of the pancreas, making it more difficult for beta cells to survive and proliferate.

### *Ob/Ob* ^*HFD*^ mice model showed elevated levels of serum IL-6 and impaired pancreatic trNK cells

**FIGURE 3:**
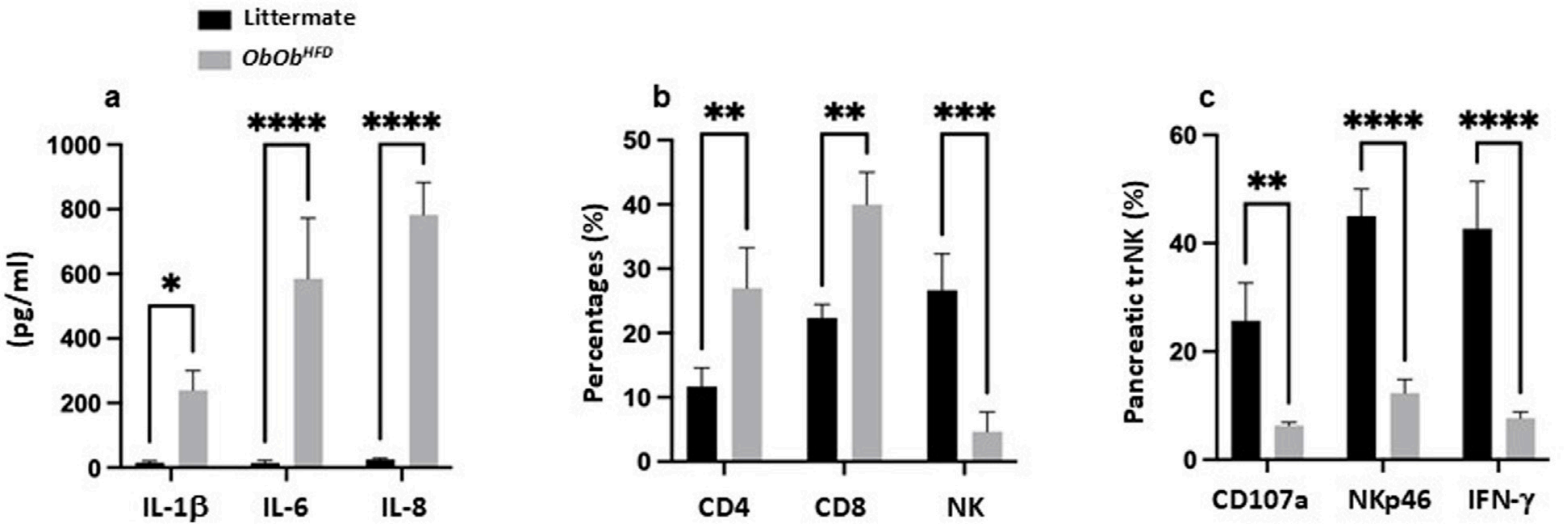
*Ob/Ob* ^*HFD*^ showed systemic inflammation and altered immune cell populations in the pancreas. **(a)** Serum levels of proinflammatory cytokines (IL‐1β, IL-6, and IL-8) were measured by ELISA (pg/mL). Flow cytometric quantification of **(b)** CD4 T cells, CD8 T cells, and NK cells (percentages) and lung trNK‐cell functionality **(c)** indicated by expression of CD107a, NKp46, and IFN‐γ. Black bars represent littermate controls; gray bars represent *Ob/Ob* ^*HFD*^ mice (n = 10) in every group. Data are expressed as averages±SD; *p < 0.05, **p < 0.01, ***p < 0.001, ****p < 0.0001 by unpaired two-tailed Student’s t-test.

To elucidate the contribution of systemic inflammation to the deterioration of β-islet cells, phenotypic alterations, and it’s possible role in changes in pancreatic immunity, we aimed to assess circulating serum pro-inflammatory cytokines IL-1β, IL-6, and IL-8 by ELISA as a reflection of their involvement in mediating immune cell fluctuations. Figure 3A demonstrate *Ob/Ob* ^*HFD*^ mice exhibiting increase in serum IL-1β (240 ± 35 pg/mL vs. 18 ± 4 pg/mL in controls; p < 0.05), IL-6 (580 ± 110 pg/mL vs. 12 ± 3 pg/mL; p < 0.0001), and IL-8 (780 ± 80 pg/mL vs. 15 ± 5 pg/mL; p < 0.0001), indicating an association of obesity and pancreatic injury with systemic inflammation. Consequently, evaluating pancreatic immune cells in the context of obesity and pancreatic injury is crucial for understanding the mechanisms driving inflammation, fibrosis, and metabolic dysfunction, ultimately paving the way for targeted therapeutic strategies to prevent or reverse pancreatic damage and improve metabolic health. For this purpose, we next assessed tissue-resident (tr) pancreatic CD4 and CD8 T cells and NK cells via flow cytometry. Figure 3B showed *Ob/Ob* ^*HFD*^ mice having elevated percentages of CD4 (27% ± 5% vs. 11% ± 3% in controls; p < 0.01) and CD8 T cells (40% ± 4% vs. 22% ± 3% in controls; p < 0.01), with a dramatic reduction in trNK cells (5% ± 4% vs. 26% ± 4% in controls; p < 0.001), suggesting T cells playing critical roles in inflammation and tissue damage while reduction in trNK cells could in part potentially impair the body’s ability to control inflammation and tissue damage effectively. Therefore, to further focus on whether reductions in trNK cells are associated with their dysregulation, we assessed their activity as mentioned in the materials and methods.

Figure 3C shows the expression of degranulation marker CD107a, activating receptor NKp46, and intracellular IFN-γ in pancreatic trNK cells. In *Ob/Ob* ^*HFD*^ mice, CD107a^+^ trNK cells were markedly reduced (6% ± 2% vs. 25% ± 4% in control; p < 0.01), as were NKp46 (17% ± 3% vs. 45% ± 5% in control; p < 0.0001) and IFN-γ subsets (8% ± 2% vs. 42% ± 9% in control; p < 0.0001) with overall reductions of 2-fold. These findings indicate a severe impairment in the cytotoxic function of trNK cells, suggesting a compromised immune response in *Ob/Ob* ^*HFD*^ mice.

### Transplanted trNK cells treated with IL-6R antagonizing antibody restored pancreatic trNK activity and attenuated pancreatic injury in a HFD mouse model

**FIGURE 4:**
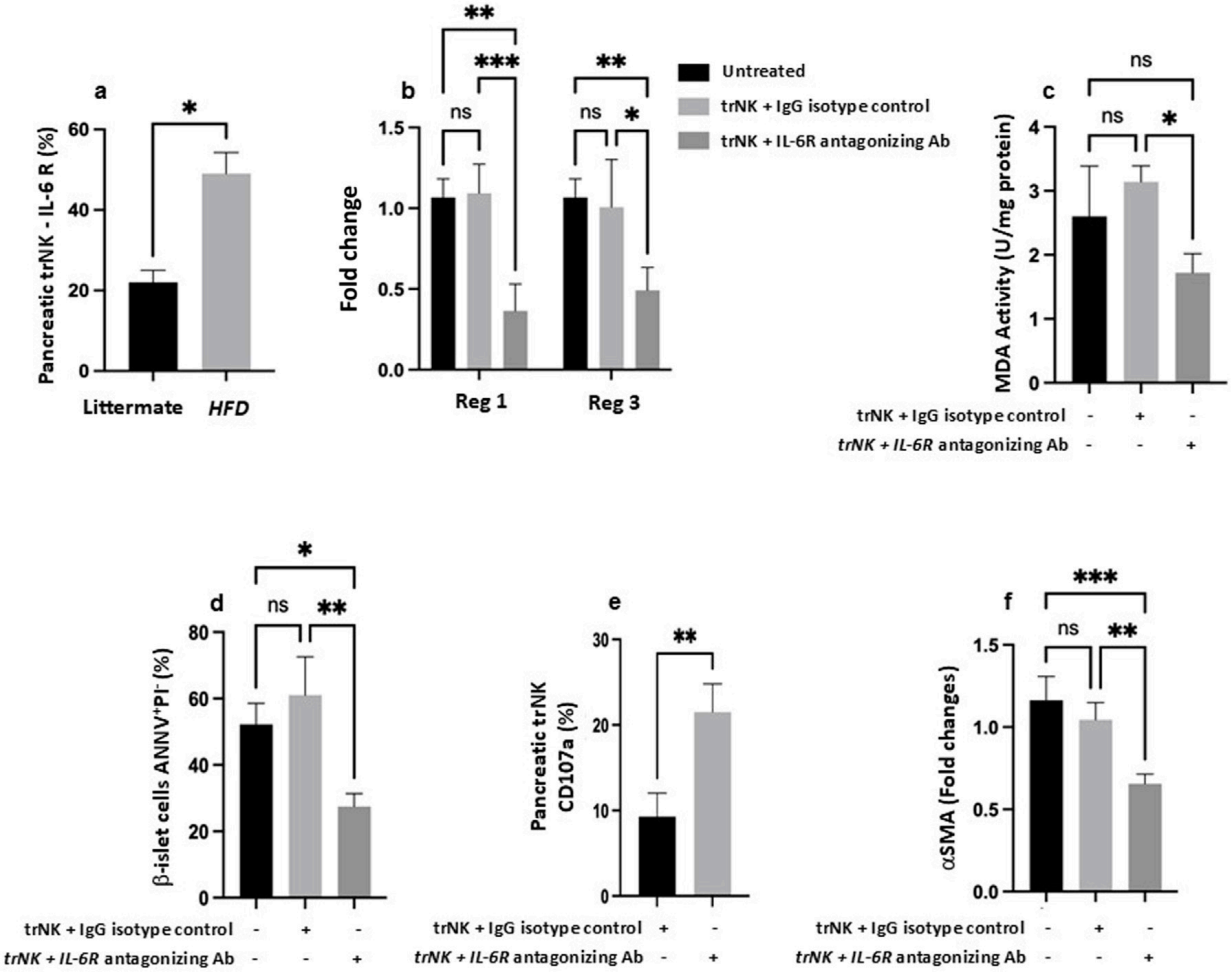
Antagonizing IL-6 receptor (R) restores pancreatic trNK cells and attenuates pancreatic injury in the MASH mice model. (a) Flow cytometric quantification of pancreatic NK cells percentages expressed IL-6R in littermate and Ob/ObHFD mice. (b) RT-PCR assessed pancreatic gene expression of Reg1 and Reg3 as fold change. (c) Pancreatic tissue MDA activity (U/mg) assessed using ELISA. Flow cytometric quantification of (d) b-islet cells apoptosis marker expressed as percentages of AnnexinV+PI- and (e) pancreatic trNK cells expressed as CD107a percentages. (f) RT-PCR gene expression of a fibrotic marker of αSMA in pancreatic tissue represented as fold change. N=6 in every group. Data are expressed as averages±SD; *p<0.05, **p<0.01, ***p<0.001, ****p<0.0001 by 2-way ANNOVA test.

Given the pivotal role of cytokines in the pathogenesis of pancreatic injury and their impact on regulating the immune response, we have assessed IL-6R expression on pancreatic trNK cells. IL-6 promotes pancreatic injury, fibrosis, and β-islet cell dysfunction. Yet, IL-6’s role in impairing trNK cell cytotoxic potential and contributing to a dysregulated immune response is not well documented. Figure 4A illustrates a significant 2.2-fold elevation in trNK IL-6R (P > 0.05) in *Ob/Ob* ^*HFD*^ mice compared to littermate controls, a result positively associated with the increase in serum IL-6 levels in these mice, as presented in Figure 3A, and inversely correlated with trNK activity markers, as shown in Figure 3C.

To strengthen the translational relevance of our findings and emphasize the contribution of IL-6 signaling to the immune modulation of obesity-associated pancreatic injury, we employed an adoptive transfer model to validate our results. We aimed to clarify the immune impact of pancreatic trNK cells in modulating pancreatic injury outcomes. To achieve this, we employed the NSG mouse strain, which is widely used for modeling immunosuppression. The immunosuppressed NSG mouse strain lacks T, B, and NK cells (Aryee et al., 2023) and was fed with HFD as mentioned in Materials and Methods, mimicking the MASH model and serving as the recipient. On the other hand, isolated pancreatic trNK cells from *Ob/Ob* ^*HFD*^ mice served as donor-transplanted cells. Figure 4B shows regenerative markers of Reg1 and Reg3 to be comparable in the pancreatic tissue of mice of both received or did not receive the trNK treated with the IgG isotype control. In parallel, NSG mice fed HFD transplanted with pancreatic trNK cells obtained from Ob/ObHFD mice and pre-treated with IL-6R antagonizing antibody exhibited a 2-fold decrease in both Reg1 and Reg3 (P<0.01). Similar patterns were achieved in pancreatic tissue MDA levels (Figure 4C) and β-islet cells apoptosis rate (Figure 4D) and showed a 1.77-fold and 2.2-fold decrease, respectively was noticed following trNK treated with the IL-6R antagonizing antibody (Figure 4C; P<0.01). Additionally, to further confirm the role of trNK cells in attenuating pancreatic injury, isolated pancreatic trNK cells treated with an IL-6R antagonizing antibody from the recipient mice and demonstrated a significantly higher expression of the NK cell activation marker CD107a (2.3 -folds) compared to mice received trNK with no IL-6R antagonizing antibody treatments (Figure 4E; P < 0.05). These findings suggest that IL-6/IL-6R modulates trNK cell activity, contributing to the alleviation of obesity-related pancreatic injury mediated by NK cells. Moreover, further phenotypic characterization of the NSG mice fed an HFD and transplanted with an IL-6R antagonizing antibody showed an amelioration in the pancreatic fibrotic profile, as evidenced by a 1.6-fold increase in α-SMA mRNA expression compared to their counterparts (Figure 4F; P<0.01). To investigate whether IL-6R signaling directly modulates STAT3 activation in pancreatic trNK cells, we performed Western blot analysis for phosphorylated STAT3 (p-STAT3) and total STAT3 in trNK cells treated *ex vivo* with either an IL-6R neutralizing antibody or an IgG isotype control. As shown in Supplementary Figure S2, trNK cells treated with IL-6R-neutralizing antibodies exhibited a marked reduction in STAT3 phosphorylation levels compared to isotype-treated controls, while total STAT3 levels remained unchanged. Quantitative densitometric analysis confirmed a significant decrease in P-STAT3/STAT3 ratio in the IL-6R antibody-treated group (*p < 0.05). These findings indicate that IL-6R antagonism effectively inhibits STAT3 signaling in pancreatic trNK cells, supporting a direct role for IL-6R in modulating their activation state. Our data clearly showed the importance of immune modulation of IL-6R on trNK cells in remodeling pancreatic tissue affected by liver injury insults, highlighting the liver-pancreas axis as a target therapy to prevent pancreatic damage, particularly β-islet cells dysfunction or loss, pancreatic fibrosis, and therefore minimize the risk of diabetes mellitus and other associated metabolic syndromes.

## Discussion

The current study extends previous research on IL-6’s role in liver steatosis and insulin resistance by highlighting its deleterious impact on the pancreas, particularly in modulating β-islet cells’ health and immune cell composition (Rohm et al., 2022; McGillicuddy et al., 2009; Donath and Shoelson, 2011). While earlier studies have focused predominantly on hepatocytes and adipose tissue, presents a novel dimension by explicitly focusing on pancreatic trNK cells, a relatively underexplored immune subset in the context of obesity-induced pancreatic injury and MASH. To the best of our knowledge, this is the first study to demonstrate that IL-6 receptor signaling directly impairs trNK cell cytotoxic function in the pancreas, contributing to β-islet cell apoptosis and fibrotic remodeling. Furthermore, although the liver-pancreas axis has been conceptually explored, our data demonstrates a unique immune-modulatory link between hepatic inflammation and pancreatic NK cell dysfunction. By showing that hepatic IL-6 spillover impacts pancreatic immunity and can be pharmacologically corrected through IL-6R antagonism, we propose a targetable inter-organ immune axis that provides a novel therapeutic framework, particularly in the context of preventing β-cell failure and diabetes progression in MASH.

Moreover, with the use of adoptive transfer of trNK cells into NSG mice (NOD-SCID IL2rγnull) mice, which lack endogenous T, B, and NK cells and subsequent reversal of pancreatic injury by IL-6R blockade provides direct mechanistic evidence that the immune dysfunction is not merely correlative but causally linked to IL-6R-mediated suppression of trNK activity. This functional rescue of NK cell cytotoxic markers, along with the simultaneous attenuation of oxidative stress and fibrosis, distinguishes our work from earlier studies that have primarily examined IL-6 in hepatocytes or adipose tissues. Additionally, our data demonstrated that treatment with an IL-6R neutralizing antibody led to a significant decrease in STAT3 phosphorylation levels in pancreatic trNK cells. This reduction was accompanied by functional restoration of trNK cell activity and attenuation of pancreatic injury. This finding is consistent with previous reports indicating that STAT3 activation impairs NK cell activation and effector function (Cacalano, 2016; Witalisz-Siepracka et al., 2022). These findings suggest that IL-6R blockade rescues trNK cell function primarily through direct inhibition of the STAT3 signaling pathway, rather than via indirect changes in the cytokine milieu.

Research interests in targeting IL-6R have shown clinical efficacy in various inflammatory diseases (Rosenberg et al., 2023; Sarfraz et al., 2021; Hossein Heydari et al., 2023; Østergaard et al., 2023; Pugliesi et al., 2023); however, its role in liver fibrosis remains complex and not fully elucidated. For instance, preclinical studies using the monoclonal antibody Tocilizumab alleviated systemic inflammation and corrected metabolic dysregulation. It also showed to paradoxically promote hepatic fibrosis under certain conditions (Gunes et al., 2023; Kou et al., 2023; Rose-John, 2017). For example, in a collagen-induced arthritis model, tocilizumab reduced inflammatory markers but induced liver collagen deposition, highlighting a dual role of IL-6 in both inflammation and tissue remodeling9. Clinical data further support this complexity. Cross-sectional studies have observed elevated fibrosis scores, such as the FIB-4 index, in patients treated with tocilizumab compared to those on other disease-modifying antirheumatic drugs (DMARDs), raising concerns about potential hepatic fibrogenesis (Fautrel et al., 2021).Additionally, pooled safety analyses from long-term trials revealed frequent but generally reversible elevations in liver enzymes, suggesting a need for ongoing hepatic monitoring (Strand et al., 2017). Case reports have also described safe use of tocilizumab in patients with pre-existing portal hypertension, but these outcomes are highly context-dependent (Amouzougan et al., 2018). Collectively, these findings underscore the importance of understanding IL-6’s tissue-specific roles and suggest that while IL-6R antagonists hold promises for modulating immune-driven fibrotic pathways, their translation into therapies for liver fibrosis must be approached with caution. Patient stratification, rigorous safety monitoring, and further mechanistic studies are essential to define the therapeutic window and minimize hepatic risk.

Our data underscores the therapeutic potential of targeting the liver-pancreas immune axis to prevent or reverse pancreatic dysfunction in metabolic disease.

## Data Availability

The original contributions presented in the study are included in the article/Supplementary Material, further inquiries can be directed to the corresponding author.

## Supplementary material

SUPPLEMENTARY FIGURE S1 Ob/ObHFD showed an increased pancreas-to-body weight ratio. Bar graph showing the significantly elevated pancreas-to-body weight ratio in Ob/ObHFD mice compared to littermate controls. Black bars represent littermate controls; gray bars represent Ob/ObHFD mice (n = 10) in every group. Data are expressed as averages ± SD; *****p* < 0.0001 by t-test.

SUPPLEMENTARY FIGURE S2 IL-6R neutralization reduces STAT3 phosphorylation in pancreatic trNK cells. Western blot analysis of phosphorylated STAT3 (P-STAT3) and total STAT3 in pancreatic trNK cells treated ex vivo with either IL-6R neutralizing antibody or isotype IgG control. Densitometric quantification of P-STAT3 levels was normalized to total STAT3 and expressed relative to the isotype control group. Bars represent averages ± SD; **p* < 0.05, unpaired two-tailed Student’s t-test; n = 5 per group.
