# Supplementary figures and images for: Antagonizing IL-6 receptor restores pancreatic tissue resident NK cells activation and ameliorates pancreatic injury in the mouse model of MASH
Source: Front Pharmacol. 2025 Jul 7;16:1611637. doi: 10.3389/fphar.2025.1611637 (PMC12277254; doi:10.3389/fphar.2025.1611637)

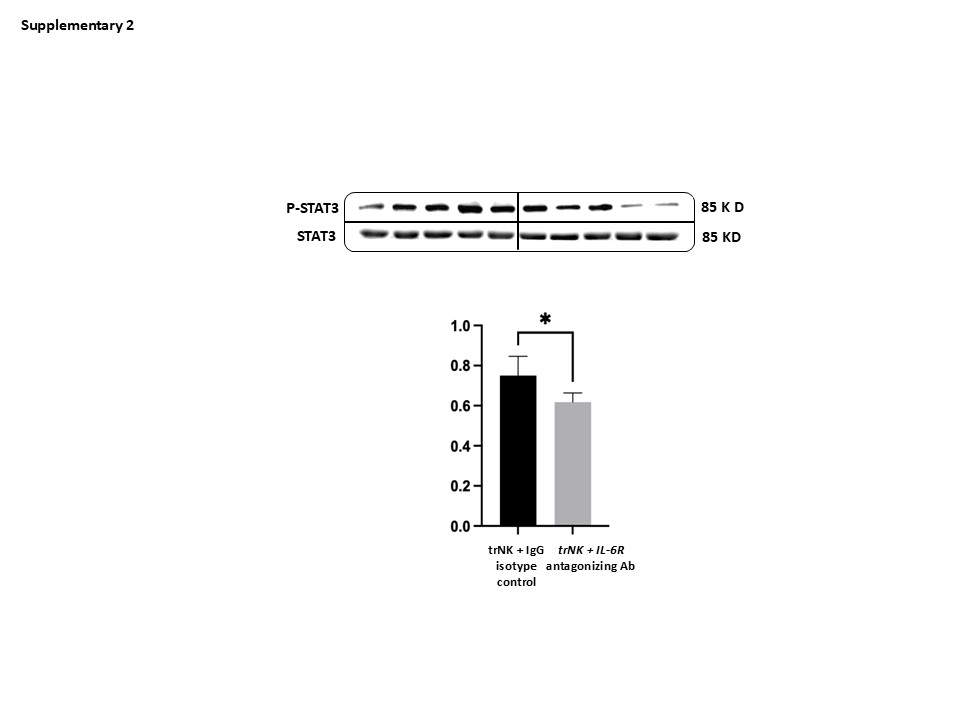

Supplement: Supplementary file 1 [file Image2.jpg]

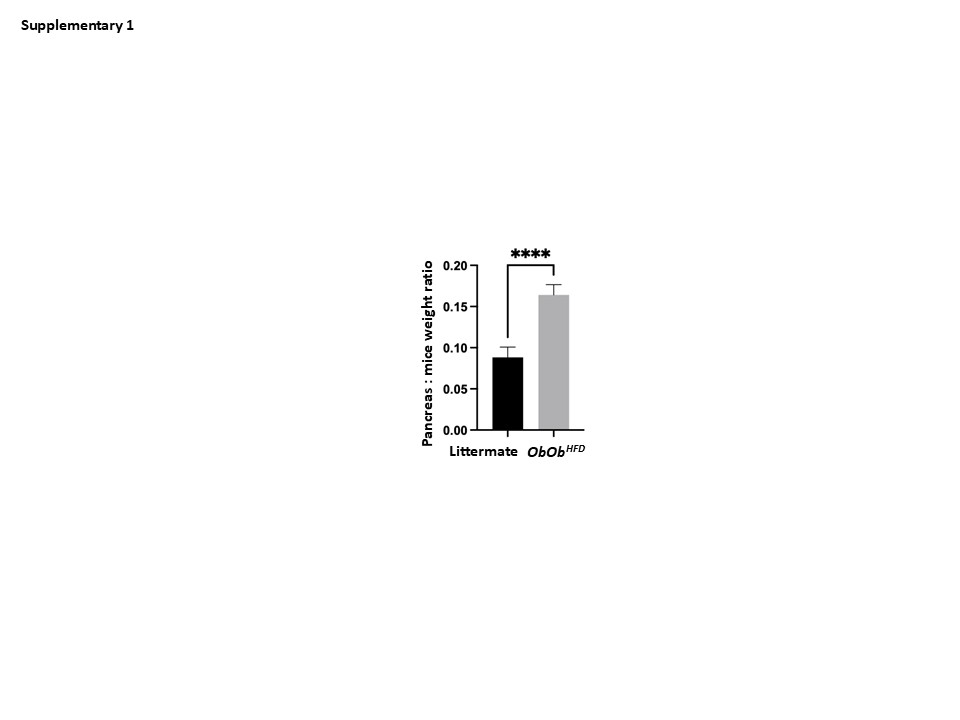

Supplement: Supplementary file 2 [file Image1.jpg]
